# Genetic Ancestry and Affinity of a Local Population in Southern Thailand Using Whole-Exome Sequencing

**DOI:** 10.3390/life16101651

**Published:** 2026-09-30

**Authors:** Pongsakorn Choochuen, Surasak Sangkhathat, Nattanan Sukpan, Kamonnat Singkhamanan, Monwadee Wonglapsuwan, Panupong Sukpan, Komwit Surachat

**Affiliations:** 1 Faculty of Medicine, Prince of Songkla University, Hat Yai 90110, Songkhla, Thailand; am_pcc1@hotmail.com (P.C.); skamonnu@medicine.psu.ac.th (K.S.); 2 Department of Research, Faculty of Medicine Siriraj Hospital, Mahidol University, Bangkok 10700, Thailand; surasak.sag@mahidol.ac.th; 3 Tak Bai Hospital, Tak Bai, Narathiwat 96110, Thailand; 4 Faculty of Science, Prince of Songkla University, Hat Yai 90110, Songkhla, Thailand; 5 Medical Education Center, Naradhiwas Rajanagarindra Hospital, Narathiwat 96000, Thailand

**Keywords:** Che He, Tai Tak Bai, Southern Thailand, whole-exome sequencing, population structure, genetic ancestry, EthSEQ

## Abstract

Population-specific genomic references remain limited for many Southeast Asian communities, particularly in Southern Thailand. We characterized the broad genetic affinity of 24 unrelated adults from Che He Subdistrict, Tak Bai District, Narathiwat Province, using whole-exome sequencing. Reads were processed against GRCh38, and population structure was assessed using reference-defined principal component analysis (PCA), ADMIXTURE, pairwise FST, EthSEQ, and KING-based relatedness analysis. A mean of 114.5 million reads per participant was retained (99.03%), with 99.98–99.99% mapping and a mean depth of 117.07× across the manufacturer-defined Agilent V8 targets. Joint genotyping identified 421,801 PASS variants. For expanded analysis, 24 Che He samples were compared with 3380 high-quality unrelated HGDP–1000 Genomes references. Group-specific missingness control retained 183,930 callable autosomal SNPs; reference-only minor-allele-frequency filtering and LD pruning retained 43,353 SNPs for projection. Che He clustered within the East Asian reference space, with its centroid closest to Cambodian across higher-dimensional summaries. Unpruned Weir–Cockerham FST was also lowest versus Cambodian (0.00256), followed by Dai (0.00892), KHV (0.00956), CDX (0.01149), and Lahu (0.01202). These results indicate relative genetic proximity within the available reference panel and should not be interpreted as definitive ancestry or ethnicity assignment.

## 1. Introduction

Human genomic studies have transformed the investigation of population history and the interpretation of disease-associated variation. However, the uneven representation of global populations in genomic databases can reduce the accuracy of ancestry inference, variant interpretation, polygenic prediction, and precision-medicine applications for underrepresented communities [1,2]. Large-scale Asian genomic initiatives, including Pan-Asian and GenomeAsia efforts, further emphasize that Asian genetic diversity is highly structured and incompletely captured by broad continental labels [3,4]. Population-specific reference resources are therefore important not only for reconstructing demographic history but also for distinguishing rare pathogenic variants from variants that are common within a particular ancestry or geographic group.

Thailand is ethnolinguistically diverse and lies at the center of Mainland Southeast Asia. Genome-wide and uniparental-marker studies have revealed genetic structure associated with geography, language family, migration, isolation, and interaction among neighboring populations [3,4,5,6,7]. The Thai Reference Exome database further demonstrated the value of sequencing local populations by cataloguing variants from more than one thousand unrelated Thai individuals and showing broad affinity to East and Southeast Asian populations [8]. Nevertheless, national or aggregated Thai datasets may not adequately represent geographically restricted communities, particularly those in Southern Thailand.

Southern Thailand extends along the Malay Peninsula and has long been connected to both Mainland and Island Southeast Asia through migration, trade, language contact, and political exchange. Previous studies using autosomal short tandem repeats reported substantial diversity among Southern Thai populations and genetic connections between Thai-Malay Muslim, Thai Buddhist, and Malaysian Malay populations [9,10]. More recent genome-wide and uniparental analyses identified South Asian-related ancestry in several populations from Thailand, including Southern Thai groups, while also demonstrating complex interactions among Kra-Dai- and Austronesian-speaking populations [6,7,11,12]. Studies of Malay populations from Peninsular Malaysia similarly indicate ancestry from multiple Asian sources [13,14]. These findings indicate that the genetic landscape of Southern Thailand cannot be represented adequately by a single national reference group.

Che He is a subdistrict of Tak Bai District in Narathiwat Province near the Thailand–Malaysia border. The name is also locally associated with the Tak Bai-speaking community. Because ethnonyms, linguistic identities, religious identities, and administrative place names are not interchangeable, this study uses the term “Che He population” operationally to refer to participants recruited from Che He Subdistrict who fulfilled predefined multigenerational residence and unrelatedness criteria. It is not intended as a definitive ethnographic classification.

In this pilot study, we used whole-exome sequencing (WES) and reference-based population-genetic analyses to characterize the broad genetic affinity of 24 Che He participants. The objectives were to determine their placement relative to global reference populations, verify sample relatedness and quality, and establish an initial genomic dataset for higher-resolution studies of population history and medically relevant variation in Southern Thailand.

## 2. Materials and Methods

### 2.1. Study Design and Participants

The present population-genomic analysis included 24 adults residing in Che He Subdistrict, Tak Bai District, Narathiwat Province, Thailand. The final cohort included 17 females (70.8%) and 7 males (29.2%). The mean age was 45.0 ± 12.1 years (median, 47.0 years; interquartile range, 34.25–49.25 years; range, 25–69 years), with age available for all participants. Sex was reported as recorded in the recruitment records. Individual-level language and self-identified ethnocultural information were not systematically recorded and were not inferred from residence, names, or genetic results. Eligibility required participants to be at least 18 years old; to report that both parents and all four grandparents had resided in the community without recent migration from another region; and to have no known biological relationship with any other participant within at least two generations. Individuals reporting a parent or grandparent from a different population group, individuals with a bleeding disorder that could increase the risk of blood collection, and individuals who declined participation were excluded. Written informed consent was obtained from all participants. The study was conducted in accordance with the Declaration of Helsinki and under the Genomics Thailand protocol approved by the Human Research Ethics Committee, Faculty of Medicine, Prince of Songkla University (REC no. 64-434-10-1; protocol no. 63-142 [CREC009/63BRm-PED]; amendment approved 7 October 2022).

### 2.2. DNA Extraction and Whole-Exome Sequencing

Peripheral blood was collected from each participant, and genomic DNA was extracted from peripheral blood leukocytes using the DNeasy Blood & Tissue Kit (Qiagen, Hilden, Germany) according to the manufacturer’s instructions. Exome libraries were prepared using the SureSelectXT HS Human All Exon V8 capture system (design ID S33266340; Agilent Technologies, Santa Clara, CA, USA). Library concentration was quantified using the Qubit dsDNA High Sensitivity Assay (Thermofisher, Waltham, MA, USA), and fragment-size distribution was assessed using the Agilent D1000 ScreenTape system (Agilent Technologies, Santa Clara, CA, USA). Sequencing was performed by Macrogen using an Illumina platform (Illumina, San Diego, CA, USA) with 151-bp paired-end reads. The provider reported use of the SureSelect V8-Post workflow.

### 2.3. Bioinformatics Workflow Overview

Bioinformatics analysis followed a staged, restartable workflow (Supplementary Figure S1). First, FASTQ integrity and read quality were checked, and adapters and low-quality bases were removed. Second, cleaned reads were aligned to GRCh38 within standardized GENCODE v47 calling intervals, followed by duplicate marking, base-quality score recalibration, BAM validation, and coverage assessment using the manufacturer-defined Agilent V8 target intervals. Third, per-sample genomic VCFs were generated with GATK HaplotypeCaller, jointly genotyped, hard-filtered, and converted to an autosomal biallelic SNP dataset for sample- and variant-level quality control, including a manufacturer-target-restricted sensitivity dataset. Finally, relatedness and internal PCA were assessed within the Che He cohort, after which shared SNPs were harmonized with reference genotypes for population-genetic analyses.

### 2.4. Raw-Read Quality Control and Preprocessing

FASTQ integrity was verified against the MD5 checksum values supplied by the sequencing provider. Raw-read quality was assessed with FastQC v0.12.1 [15], and reports were aggregated with MultiQC v1.35 [16]. Adapter trimming and quality filtering were performed with fastp v1.3.6 [17], with paired-end adapter detection and overlap-based base correction enabled. Bases with Phred quality scores below 20 were treated as unqualified; read pairs were discarded when more than 40% of bases were unqualified or when either mate was shorter than 50 bp after processing. Fastp JSON and HTML reports were retained, trimmed FASTQ files were checked for gzip integrity, and post-trimming quality was reassessed with FastQC and MultiQC.

### 2.5. Reference Genome and Known-Sites Resources

Cleaned reads were processed against the Broad Institute GRCh38 human reference genome. The resource bundle comprised the GRCh38 FASTA sequence, FASTA index, sequence dictionary, dbSNP build 138, the Mills and 1000 Genomes gold-standard indel dataset, and the GRCh38 known-indel resource [18,19]. The reference was indexed for alignment with BWA-MEM2 v2.2.1 [20,21] and for sequence manipulation with SAMtools v1.23.1 [22,23].

### 2.6. Definition of Capture Targets and Genomic Calling Regions

Manufacturer-defined capture targets were obtained from the GRCh38 BED files for the SureSelectXT HS Human All Exon V8 design (design ID S33266340; Agilent Technologies). The primary manufacturer target intervals were restricted to canonical GRCh38 chromosomes, sorted, and merged, producing a target span of 35.130788 Mb. GENCODE v47 protein-coding exon intervals [24] extended by 100 bp on both sides, which had been used for base-quality recalibration and variant calling, were retained as the broader calling space (154.047495 Mb) and were not treated as equivalent to the capture target. This calling space encompassed 34.816905 Mb (99.107%) of the manufacturer-defined target bases [25].

### 2.7. Read Alignment and BAM Processing

Trimmed paired-end reads were aligned to GRCh38 with BWA-MEM2 v2.2.1, which implements a performance-optimized BWA-MEM algorithm [20,21,26]. Sample-specific read-group information was assigned during alignment. Alignments were coordinate-sorted, converted to BAM format, indexed, and validated with SAMtools v1.23.1 [22,23]. PCR and optical duplicates were marked with GATK MarkDuplicates (GATK v4.6.2.0; Picard v3.4.0), and duplicate metrics were retained for downstream quality assessment [27,28].

### 2.8. Base Quality Score Recalibration

Base-quality score recalibration was performed with GATK BaseRecalibrator and ApplyBQSR according to GATK best-practice principles [27,28]. Known sites comprised dbSNP build 138, the GRCh38 known-indel dataset, and the Mills and 1000 Genomes gold-standard indel dataset. Recalibration was restricted to the padded GENCODE exon intervals. Recalibrated BAM files were indexed and validated before variant calling.

### 2.9. Alignment and Manufacturer-Target Coverage Assessment

Post-alignment quality assessment integrated SAMtools v1.23.1 [22,23], GATK duplicate metrics [27,28], mosdepth v0.3.14 [29], and MultiQC v1.35 [16]. Recorded metrics included total aligned reads, overall mapping rate, properly paired read rate, duplicate rate, insert size, and depth across the exact manufacturer-defined Agilent V8 target intervals. Target-coverage summaries included mean depth and the proportions of target bases covered at ≥10×, ≥20×, ≥30×, and ≥50×. Coverage across the broader GENCODE-derived calling space was retained only as a secondary technical description and was not interpreted as manufacturer-defined on-target coverage.

### 2.10. Per-Sample Germline Variant Calling and Joint Genotyping

Germline variants were called independently for each participant with GATK HaplotypeCaller v4.6.2.0 in genomic VCF mode with reference-confidence output [27,28]. Calling was restricted to the padded GENCODE exon intervals. The 24 per-sample gVCFs were combined and jointly genotyped with GATK GenotypeGVCFs across the same intervals to produce the cohort-level callset.

### 2.11. Variant Filtering

Single-nucleotide polymorphisms and small insertions/deletions were separated and hard-filtered independently with GATK v4.6.2.0 [27,28]. SNP filters were QD < 2.0, QUAL < 30.0, SOR > 3.0, FS > 60.0, MQ < 40.0, MQRankSum < −12.5, or ReadPosRankSum < −8.0. INDEL filters were QD < 2.0, QUAL < 30.0, FS > 200.0, or ReadPosRankSum < −20.0. Only variants labelled PASS or without a filter label were retained, after which the filtered SNP and INDEL datasets were combined.

### 2.12. Variant- and Sample-Level Quality Control

Variant- and sample-level quality control was performed with BCFtools v1.23.1 and PLINK 2.0 v2.0.0-a.6.9LM [23,30]. The population-genetic dataset was restricted to autosomal biallelic SNPs with standard A, C, G, or T alleles. Because exome-derived datasets can show uneven locus sharing, missingness thresholds were evaluated empirically. The primary dataset retained variants with genotype missingness ≤ 20%, samples with genotype missingness ≤ 30%, and variants with minor allele frequency ≥ 1%. To evaluate sensitivity to target definition, PASS calls were additionally restricted to the exact manufacturer-defined Agilent V8 targets before autosomal biallelic SNP selection and application of the same WES-adjusted quality-control criteria. Summary statistics included SNP and INDEL counts, transition/transversion ratio, per-sample missingness, allele frequencies, observed and expected heterozygosity, and inbreeding coefficients.

### 2.13. Relatedness and Kinship Analysis

Pairwise relatedness was assessed with the KING-robust method implemented in PLINK 2.0 [30,31]. Autosomal SNPs passing WES-adjusted sample and variant QC were LD-pruned using a 200-variant window, a 50-variant step, and an r^2^ threshold of 0.2. Because the cohort comprised fewer than 50 individuals, allele-frequency estimates from the pruned dataset were supplied to address PLINK small-sample warnings. Kinship coefficients were interpreted using standard KING thresholds for duplicate/monozygotic, first-degree, second-degree, and third-degree relationships, with the primary aim of identifying unexpected close relatives.

### 2.14. Principal Component Analysis

Internal principal component analysis was performed in PLINK 2.0 using the LD-pruned autosomal SNP dataset from the Che He cohort [30,32]. The first 20 principal components were calculated to assess sample clustering, dispersion, and potential outliers. This analysis was used for within-cohort quality assessment and was not interpreted as population-ancestry assignment.

### 2.15. Expanded HGDP–1000 Genomes Reference-Panel Harmonization and Projected PCA

For expanded reference-panel analysis, autosomal biallelic Che He SNPs were harmonized by chromosome, position, reference allele, and alternate allele with gnomAD v3.1.2 HGDP–1000 Genomes genotypes [33]. The reference set comprised 3380 high-quality unrelated individuals (2510 from 1000 Genomes and 870 from HGDP), spanning 78 populations and seven broad genetic regions. Exact allele-matched harmonization yielded 297,205 shared SNPs across 3404 individuals. To limit cross-platform differential missingness, variants were retained only when missingness was ≤5% separately in 1000 Genomes, HGDP, and Che He, leaving 183,930 callable SNPs. PCA axes were defined without using Che He genotypes: reference individuals alone were filtered at minor allele frequency ≥ 1% (145,291 SNPs), LD-pruned using a 200-variant window, 50-variant step, and r^2^ threshold of 0.2 (43,353 SNPs), and used to estimate 20 principal components and allele weights. All 3404 individuals were then scored against the fixed reference frequencies and allele weights without mean imputation. Population-centroid distances were summarized over PC1–4, PC1–10, and PC1–20; these distances describe relative proximity within this reference panel, not ancestry proportions or ethnic identity.

### 2.16. Model-Based Ancestry Analysis with ADMIXTURE

Model-based ancestry analysis was performed with ADMIXTURE v1.3.0 [34] using the merged Che He–1000 Genomes dataset. Autosomal biallelic SNPs were filtered at genotype missingness ≤ 5% and minor allele frequency ≥ 1%, then LD-pruned in PLINK 2.0 with a 200-variant window, 50-variant step, and r^2^ threshold of 0.2 [30]. ADMIXTURE was run for K = 2–10 with 10-fold cross-validation. The lowest cross-validation error was used as a descriptive summary, while adjacent K values were examined because small differences in cross-validation error may not identify a uniquely resolved model. Components were interpreted as reference-panel patterns rather than direct estimates of named ethnic ancestry.

### 2.17. Pairwise FST Analysis

Pairwise population differentiation between the Che He cohort and each of the 78 populations represented in the expanded HGDP–1000 Genomes panel was estimated in PLINK 2.0 using the Weir–Cockerham FST estimator [35]. The primary analysis used all 183,930 autosomal biallelic SNPs that passed the ≤5% missingness requirement separately in 1000 Genomes, HGDP, and Che He. These markers were analyzed before LD pruning and without a combined-sample minor-allele-frequency threshold. Final estimates were calculated as ratios of averages, and uncertainty was summarized with block-jackknife standard errors using blocks of 1000 variants. Depending on nonmissing observations in each population pair, 179,559–183,159 SNPs contributed to the primary estimates. To assess capture-target ascertainment, the analysis was repeated using 54,806 primary-callable SNPs that overlapped the exact manufacturer-defined Agilent SureSelectXT HS Human All Exon V8 target-restricted Che He QC dataset. Concordance between the primary and target-restricted estimates was evaluated using Pearson correlation of FST values and Spearman correlation of population ranks.

### 2.18. Identification and Annotation of Reference-Panel-Absent Recurrent Variants

To address recurrent variation not represented in the expanded comparison panel, autosomal biallelic Che He variants were compared with the exact GRCh38 REF/ALT allele keys in the HGDP–1000 Genomes reference dataset. Candidate absences were reconciled against the exact source release after multiallelic normalization; variants recovered from source multiallelic records were removed. For the remaining candidates, carrier genotypes were reassessed in the original cohort VCF and retained as high confidence when depth was at least 10, genotype quality was at least 20, heterozygous allele balance was 0.20–0.80, or homozygous-alternate allele balance was at least 0.80. Variants carried by at least two participants were considered recurrent, and those carried by at least three were prioritized for exploratory annotation. Consequences were obtained from the Ensembl Variant Effect Predictor REST service on GRCh38 [36]. MANE/canonical annotations and the most severe transcript consequence were reported separately, and clinical colocations were accepted only for exact biallelic allele matches. Throughout, “reference-panel-absent” denotes absence from this specific HGDP–1000 Genomes panel and release, not global novelty, population specificity, or pathogenicity.

### 2.19. EthSEQ Ancestry Analysis

Broad genetic affinity was additionally assessed with EthSEQ v3.0.1, an R v4.4.3 package developed for ethnicity annotation from whole-exome and targeted-sequencing genotype data [37]. The cohort VCF was analyzed with ethseq.Analysis() and the 1000 Genomes reference model, which represents African, admixed American, East Asian, European, and South Asian superpopulations [18]. SNPs shared between the target and reference datasets were used for PCA; 5972 SNPs were retained in the preliminary EthSEQ visualization. The “INSIDE” and “CLOSEST” labels were interpreted as relative positions in PCA space, and the reported relative contributions were treated as descriptive affinity estimates rather than formal demographic admixture proportions.

### 2.20. Computational Resources and Reproducibility

The analysis was conducted on a Linux workstation, and the workflow was implemented as a restartable Bash pipeline with software managed in the WES-complete Conda environment. Independent sample-level tasks were scheduled with GNU Parallel release 20260622 [38]. Executable versions were FastQC v0.12.1, fastp v1.3.6, MultiQC v1.35, BWA-MEM2 v2.2.1, SAMtools v1.23.1, BCFtools v1.23.1, HTSlib/tabix v1.23.1, GATK v4.6.2.0 (Picard v3.4.0), BEDTools v2.31.1, mosdepth v0.3.14, PLINK 2.0 v2.0.0-a.6.9LM, ADMIXTURE v1.3.0, VCFtools v0.1.17 [39], and EthSEQ v3.0.1. The complete computational workflow is shown in Supplementary Figure S1.

## 3. Results

### 3.1. Study Cohort and Sequencing Output

All 24 participants included in the analysis met the multigenerational residence and recruitment criteria. The cohort included 17 females (70.8%) and 7 males (29.2%), with a mean age of 45.0 ± 12.1 years (median, 47.0 years; range, 25–69 years) (Table 1). The sequencing provider generated 16.27–19.28 Gb of raw sequence per sample, corresponding to 107.7–127.7 million reads per participant. Raw sequence quality was consistently high, with Q30 values ranging from 92.1% to 93.7%. After fastp preprocessing, a mean of 114.5 million reads per sample was retained (range, 106.6–126.2 million), corresponding to a mean read-retention rate of 99.03% (range, 98.86–99.31%), and the GC content of the retained paired reads ranged from 47.1% to 49.6%. Post-filtering Q30 values remained high across all samples, and no sample was excluded at the raw-read QC stage. Complete raw-read and preprocessing metrics are provided in Supplementary Table S1.

### 3.2. Alignment Quality and Manufacturer-Defined Agilent V8 Target Coverage

All 24 preprocessed paired-end datasets were successfully aligned to the GRCh38 reference genome and converted into final recalibrated BAM files (Table 2). The final BAM files contained 65.1–89.1 million reads per sample (mean, 76.9 million). Mapping rates were uniformly high across the cohort (99.98–99.99%; mean, 99.99%), and the proportion of properly paired reads ranged from 97.61% to 99.45% (mean, 98.73%). Picard duplicate rates ranged from 11.98% to 19.31% (mean, 15.42%), indicating consistent library complexity across samples. The mean insert size was 254.3 bp (range, 240.0–264.7 bp). Sample-level alignment metrics are provided in Supplementary Table S2.

Coverage was recalculated using the manufacturer-defined GRCh38 target intervals for the SureSelectXT HS Human All Exon V8 design (Table 2). These target regions spanned 35.130788 Mb. The broader GENCODE v47 protein-coding exon plus 100-bp calling space spanned 154.047495 Mb and encompassed 34.816905 Mb (99.107%) of the manufacturer target bases, confirming near-complete target inclusion while remaining substantially broader than the capture design (Supplementary Table S16). Across the 24 samples, mean target depth was 117.07× (median, 117.75×; range, 99.23–138.34×). The mean proportions of target bases covered at ≥10×, ≥20×, ≥30×, and ≥50× were 98.45%, 97.93%, 97.27%, and 94.82%, respectively. Summary manufacturer-target coverage metrics are provided in Supplementary Table S3.

### 3.3. Sample Identity, Relatedness, and Cohort Quality

All 24 participants were retained after WES-adjusted PLINK filtering (Table 3 and Table 4). Before locus-level filtering, sample-level genotype missingness across the autosomal biallelic SNP dataset ranged from 15.05% to 19.88% (mean, 17.80%). After applying variant missingness ≤ 20%, sample missingness ≤ 30%, MAF ≥ 1%, and LD pruning, 16,185 SNPs were retained for internal PCA, heterozygosity, and kinship analysis. Missingness in the pruned dataset was low, ranging from 1.12% to 1.80% per sample (mean, 1.50%). Variant filtering and LD-pruning summaries are provided in Supplementary Table S4, and sample-level missingness and heterozygosity metrics are provided in Supplementary Table S5.

### 3.4. Variant Discovery and Cohort Callset

Joint genotyping followed by SNP/INDEL hard filtering generated a final PASS cohort callset containing 421,801 variants across 24 participants (Table 3). The callset comprised 360,916 SNPs and 60,885 indels, including 14,640 multiallelic sites. The SNP transition/transversion ratio was 2.08. For downstream PLINK-based analysis, the dataset was restricted to autosomal biallelic SNPs with standard nucleotide alleles, resulting in 351,623 SNPs before missingness and frequency filtering and 226,348 SNPs after WES-adjusted QC. Detailed callset and PLINK QC summaries are provided in Supplementary Table S4.

Restriction of the PASS cohort calls to the manufacturer-defined Agilent V8 targets yielded 69,512 variants, including 67,311 SNPs and 2201 indels. Restriction to autosomal biallelic SNPs retained 65,764 variants before quality control and 63,632 after WES-adjusted quality control; all 24 individuals were retained. Post-QC sample-level missingness in the target-restricted SNP dataset was low (mean, 0.0779%; median, 0.0739%; range, 0.0456–0.1666%). This dataset was retained for sensitivity analyses of target-definition effects on the population-genetic results (Supplementary Table S17).

KING-robust pairwise kinship coefficients across the 276 participant pairs ranged from −0.1356 to 0.0002 (Table 4; Supplementary Table S6). No pair approached the expected threshold for third-degree or closer relationships, supporting the recruitment criterion that participants were not close biological relatives. PLINK heterozygosity-based inbreeding coefficients ranged from −0.0163 to 0.1324 (mean, 0.0111; Supplementary Table S5); one participant showed the highest F estimate and should be monitored in reference-panel analyses, but no sample was excluded at this stage. Internal cohort PCA coordinates are provided in Supplementary Table S7 and visualized in Supplementary Figure S2.

### 3.5. Predominant Affinity to the East Asian Reference Group

EthSEQ classified 23 of the 24 participants (95.8%) as ‘CLOSEST’ to the East Asian superpopulation, and one participant, C03_2E1 (4.2%), was positioned ‘INSIDE’ the East Asian convex boundary (Table 5). No participant was assigned primarily to the African, admixed American, European, or South Asian reference group.

### 3.6. East Asian–South Asian Orientation in Principal-Component Space

For the 23 samples labelled ‘CLOSEST’ to East Asian references, the EthSEQ-reported East Asian relative contribution ranged from 68.94% to 83.84%, while the South Asian relative contribution ranged from 16.16% to 31.06%. These values describe sample placement in PCA space and are not equivalent to ancestry proportions from model-based methods. The preliminary PCA positioned the study samples adjacent to the East Asian reference region with displacement in the direction of the South Asian reference group. Reference-panel PCA, ADMIXTURE, and FST analyses are presented below as the primary population-genetic comparisons. The within-cohort PCA is provided as Supplementary Figure S2.

### 3.7. Expanded HGDP–1000 Genomes Reference-Defined PCA Projection

Exact harmonization between the Che He and expanded HGDP–1000 Genomes datasets identified 297,205 autosomal biallelic SNPs in 3404 individuals. Before group-specific variant filtering, mean sample missingness was 0.5353% in 1000 Genomes, 0.7576% in HGDP, and 16.3884% in Che He; the latter reflects cross-panel locus availability and is distinct from the 0.0779% mean missingness observed in the manufacturer-target-restricted Che He sensitivity dataset. Requiring variant missingness ≤ 5% within each of the three groups retained 183,930 SNPs. In the resulting callable dataset, mean sample missingness was 0.0272% for 1000 Genomes, 0.1525% for HGDP, and 0.1817% for Che He. Reference-only MAF filtering and LD pruning retained 43,353 SNPs for PCA projection.

**Figure 1:**
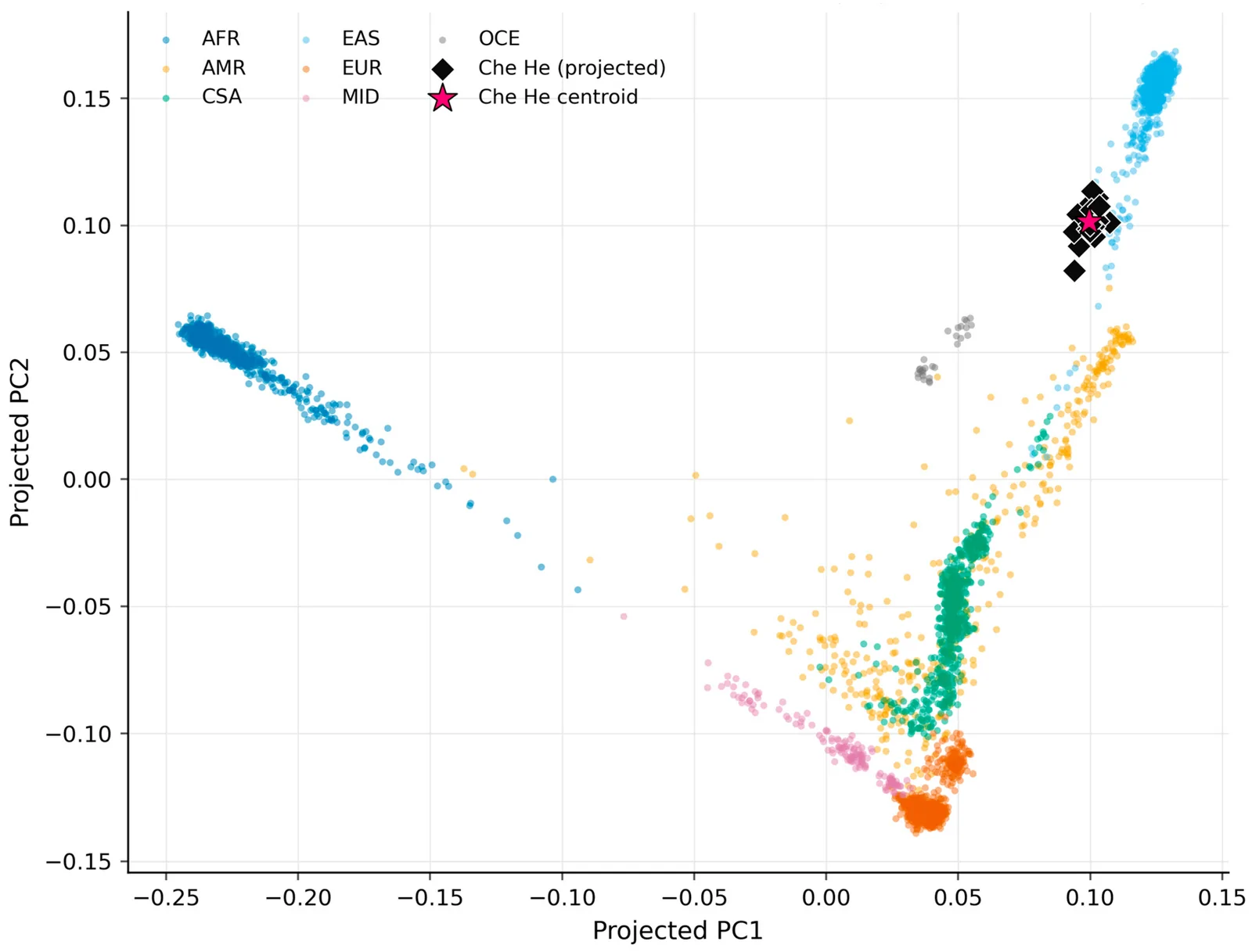
Global HGDP–1000 Genomes reference-defined PCA. Axes were learned from 3380 high-quality unrelated reference individuals using 43,353 reference-filtered and LD-pruned SNPs; 24 Che He samples (black diamonds) were subsequently projected, with the cohort centroid shown by a magenta star. Colors denote broad reference genetic regions.

**Figure 2:**
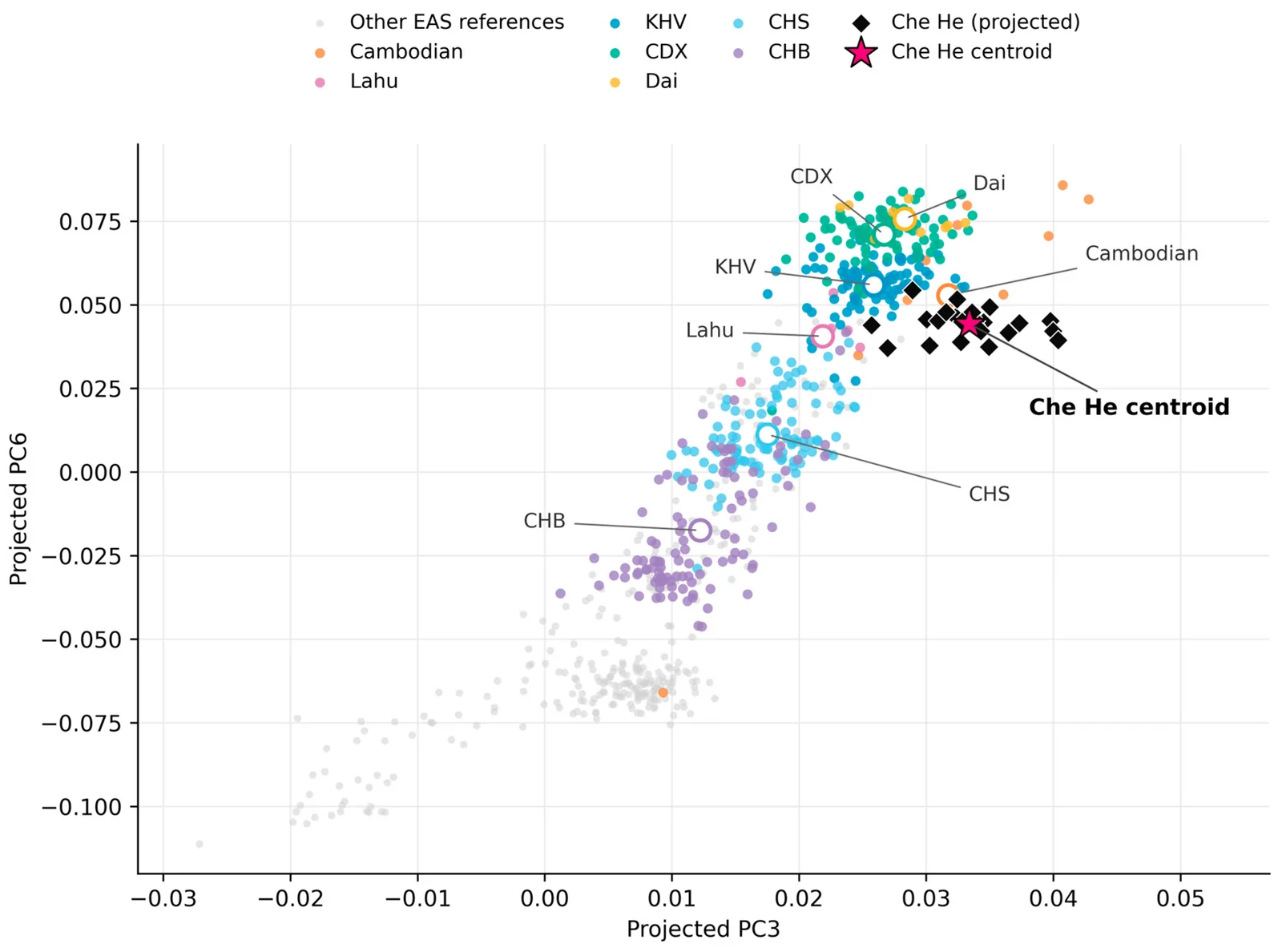
East Asian-focused view of the reference-defined PCA projection (PC3 versus PC6). Che He samples are shown as black diamonds and the cohort centroid as a magenta star. Selected comparison populations are highlighted; proximity represents position within the available reference panel rather than ancestry or ethnicity assignment.

Projection onto axes learned exclusively from the 3380 reference individuals placed all 24 Che He samples within the East Asian reference space (Figure 1 and Figure 2; Table 6). The Che He centroid was closest to the HGDP Cambodian centroid when distances were summarized over PC1–4, PC1–10, or PC1–20. In the PC1–10 and PC1–20 summaries, Lahu and KHV were the next closest centroids. Rankings based on only two or four components were less stable for several other populations, so interpretation emphasized consistency across higher-dimensional summaries and was limited to relative proximity among the available reference populations.

### 3.8. Preliminary 1000 Genomes-Only ADMIXTURE Analysis

**Figure 3:**
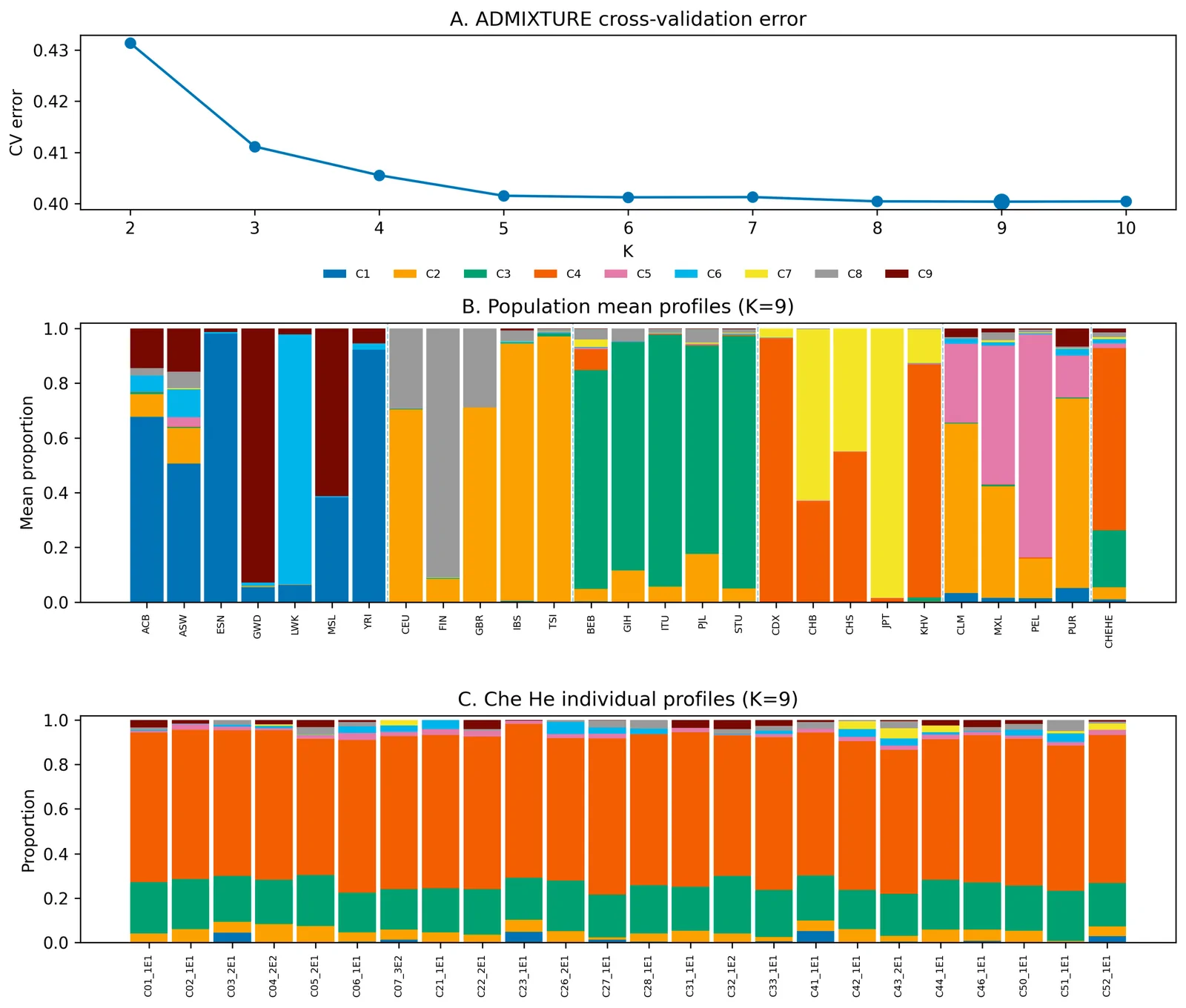
ADMIXTURE cross-validation and ancestry-component profiles for the merged Che He and 1000 Genomes dataset. (**A**) Cross-validation error for K = 2–10, with the lowest value at K = 9. (**B**) Population mean component profiles for the 1000 Genomes populations and Che He. (**C**) Individual-level ADMIXTURE profiles for the 24 Che He participants at K = 9. Components are model-derived and should be interpreted as relative reference-panel patterns rather than direct labels of ethnic ancestry. In panels (**B**,**C**), the colors represent the nine inferred ancestry components at K = 9: C1, blue; C2, gold; C3, green; C4, orange; C5, pink; C6, light blue; C7, yellow; C8, gray; and C9, dark red. The same color scheme is used in both panels.

ADMIXTURE was applied to the LD-pruned merged reference dataset containing 2572 individuals and 43,328 SNPs (Table 7; Figure 3). Cross-validation error decreased substantially from K = 2 to K = 5 and continued to decline more gradually through higher K values. The lowest cross-validation error was observed at K = 9 (CV error = 0.40039), with very similar values at K = 8 (0.40045) and K = 10 (0.40044), indicating that the cross-validation curve is effectively flat across K = 8 to K = 10 and that no single K should be treated as uniquely optimal. ADMIXTURE cross-validation errors are provided in Supplementary Table S12.

At K = 9, Che He individuals showed a broadly consistent component profile across the 24 samples (Figure 3; Supplementary Table S13). In component space, the Che He mean profile was closest to KHV and CDX among the available 1000 Genomes populations (Supplementary Table S14), supporting the PCA-based observation that the cohort lies within an East Asian-related region of the reference-panel space. However, because 1000 Genomes does not include Thai or Malay Peninsula population references, the ADMIXTURE output is best interpreted as a reference-panel comparison rather than a definitive ancestry assignment.

### 3.9. Expanded-Reference Unpruned Pairwise FST Analysis

**Figure 4:**
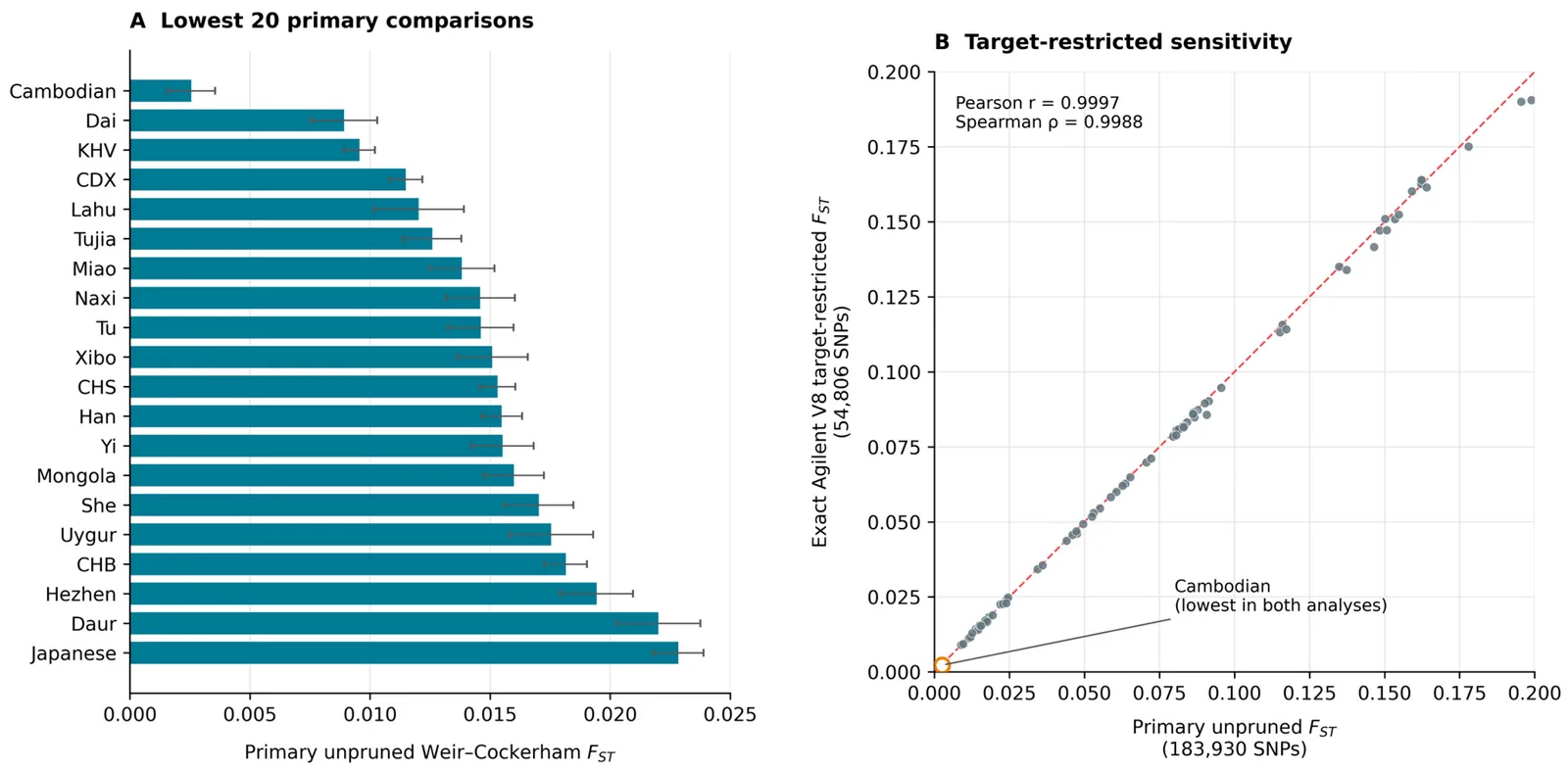
Expanded-reference unpruned pairwise FST and exact Agilent V8 target-restricted sensitivity analysis. (**A**) The 20 lowest primary Weir–Cockerham FST estimates among 78 comparisons, calculated from the 183,930-SNP callable dataset before LD pruning and without a combined-sample MAF threshold. Error bars show estimate ± 1.96 block-jackknife standard errors. (**B**) Comparison of primary estimates with estimates from 54,806 callable SNPs overlapping the exact manufacturer-defined Agilent V8 target-restricted dataset. The dashed line indicates equality. Agreement was high across all populations (Pearson r = 0.9997; Spearman rank correlation rho = 0.9988). Cambodian had the lowest FST in both analyses. Population labels denote sampled reference groups and do not imply ancestry or ethnicity assignment.

Across the 78 expanded-reference populations, the lowest unpruned Weir–Cockerham FST was observed between Che He and Cambodian (0.00256; approximate 95% interval based on the block-jackknife SE, 0.00157–0.00355; 179,678 contributing SNPs), followed by Dai (0.00892), KHV (0.00956), CDX (0.01149), and Lahu (0.01202) (Table 8; Figure 4). As a robustness check, restricting the analysis to the 54,806 callable SNPs within the manufacturer-defined Agilent V8 target preserved the same five leading populations in the same order and was highly concordant with the primary estimates (Supplementary Table S15). The agreement between the primary FST analysis and the reference-defined PCA supports Cambodian as the closest sampled comparison population, while representing relative proximity within the available panel rather than ancestry or ethnic identity.

Together, the main figures and tables summarize the analytical workflow, cohort sequencing and variant quality, internal relatedness, reference-panel PCA, ADMIXTURE, and pairwise FST results (Supplementary Figures S1 and S2 and Figure 1, Figure 2, Figure 3 and Figure 4; Table 1, Table 2, Table 3, Table 4, Table 5, Table 6, Table 7 and Table 8). Detailed and summary supporting data are provided in Supplementary Tables S1–S18, and the complete bioinformatics workflow is provided in Supplementary Figure S1.

### 3.10. Reference-Panel-Absent Recurrent-Variant Analysis

Exact allele comparison initially identified 25,340 Che He variants absent from the harmonized reference dataset. Exact-source multiallelic reconciliation recovered 2089 of these, leaving 23,251 validated reference-panel-absent candidates. Genotype-level filtering retained at least one high-confidence carrier for 16,816 variants; 1524 were recurrent in at least two participants, and 169 carried by at least three participants were annotated (Supplementary Table S18). Among these 169 candidates, MANE/canonical transcript selection classified 1 as HIGH impact, 31 as MODERATE, 25 as LOW, and 112 as MODIFIER; variant-level most-severe assessment identified 3 HIGH- and 34 MODERATE-impact candidates. In total, 118 candidates had a known variant identifier. Exact-biallelic clinical colocations were present for 11 variants (5 likely benign and 6 of uncertain significance), with none classified as pathogenic or likely pathogenic. These findings provide a quality-controlled follow-up list rather than evidence of globally novel or Che He-specific variants.

## 4. Discussion

This pilot study provides an initial exome-based characterization of genetic affinity and within-cohort genomic quality in 24 participants from Che He Subdistrict in the southern border region of Thailand. The completed WES processing workflow produced high mapping rates, high manufacturer-defined target coverage, and a final hard-filtered cohort callset of 421,801 variants. Restriction to the exact Agilent V8 targets yielded 63,632 autosomal biallelic SNPs after WES-adjusted quality control, with low sample-level missingness and retention of all 24 participants. After WES-adjusted PLINK filtering of the broader callset, 16,185 LD-pruned autosomal SNPs were available for internal PCA, heterozygosity, and relatedness assessment. These results support the technical suitability of the dataset for reference-panel population-genetic analyses and target-definition sensitivity assessment.

The expanded reference-defined PCA reduces cohort-driven axis distortion by learning allele frequencies, LD pruning, and component weights exclusively from 3380 reference individuals before projecting Che He. Across multiple higher-dimensional summaries, the Che He centroid showed its closest relative proximity to Cambodian, followed by Lahu and KHV. This refines the earlier 1000 Genomes-only comparison by adding 870 HGDP individuals and regional populations absent from the initial panel. It does not establish direct descent or self-identified ethnicity, and population labels should be understood as labels of the sampled reference groups.

The ADMIXTURE analysis further supports this interpretation while reinforcing the need for caution. Cross-validation error reached its lowest value at K = 9, but K = 8–10 differed only marginally, and the inferred components were estimated from a reference panel lacking Thai and Malay Peninsula populations. The Che He component profile was most similar to KHV and CDX population means among the available 1000 Genomes references. In the expanded unpruned FST analysis, Cambodian showed the lowest differentiation, followed by Dai, KHV, CDX, and Lahu. A target-restricted sensitivity analysis further confirmed that this relative pattern was not driven by the inclusion of off-target callable loci. The convergence of reference-defined PCA and expanded-reference FST supports relative proximity to mainland Southeast and East Asian reference groups, but does not establish direct descent, ancestry proportions, or self-identified ethnicity [18,19,40].

Predominant East Asian affinity is broadly consistent with previous studies of Thai populations. The T-REx project found Thai exomes to be most similar to East and Southeast Asian populations [8], while genome-wide studies of ethnolinguistic groups in Thailand and Laos demonstrated substantial East Asian-related ancestry combined with population-specific drift and interaction [5,6]. The Che He findings therefore fit within the wider genetic landscape of Mainland Southeast Asia while highlighting local variation that may be obscured when Thai populations are analyzed as a single national group.

The South Asian-directed signal is plausible in the regional context, but its magnitude should be interpreted cautiously. Genome-wide studies have detected South Asian-related ancestry in several populations from Thailand, including Southern Thai groups [6,11,12]. Studies of Malay populations from the Malay Peninsula and neighboring regions similarly indicate ancestry from multiple Asian sources [13,14]. Nevertheless, the current EthSEQ analysis cannot identify a specific source population, distinguish shared ancestry from recent gene flow, or estimate the timing of admixture. Formal admixture statistics, such as f3- or f4-based tests or qpAdm, were beyond the scope of this pilot and would be required to determine whether the South Asian-oriented signal reflects genuine gene flow rather than shared drift or residual technical variation in the merged dataset.

The study has potential relevance for medical genomics because population-specific allele frequencies can improve filtering of candidate variants and reduce the risk that locally common variants are misclassified as rare or pathogenic. However, this cohort is not large enough to establish reliable clinical allele frequencies. It should instead be viewed as a pilot dataset supporting future expansion of regional genomic references.

The exploratory reference-panel-absent screen further illustrates the importance of transcript-aware and genotype-aware interpretation. The sole MANE/canonical HIGH-impact candidate was a TONSL splice-donor variant observed in three participants, whereas two additional HIGH-impact consequences occurred only on alternative transcripts. Such observations warrant orthogonal validation and broader frequency assessment, but they do not establish clinical relevance. Consistent with this caution, none of the exact-biallelic clinical colocations was pathogenic or likely pathogenic, and the two locally clustered candidates had relatively low median allele balance.

Several limitations require emphasis. First, the sample size was small and participants were recruited from a single locality. Individual-level language and self-identified ethnocultural information were not systematically recorded; therefore, the geographic recruitment label must not be interpreted as a linguistic or ethnic classification. Second, whole-exome sequencing samples an assay-defined, coding-enriched subset of the genome and remains susceptible to marker-ascertainment and cross-platform effects. Group-specific missingness control was therefore necessary because initial cross-panel locus availability differed, particularly for Che He. Although post-filter missingness was low, this does not remove the inherent ascertainment limitations of WES. Third, the available reference panel still lacks dense sampling from Southern Thailand and the Malay Peninsula; proximity to Cambodian, Dai, Lahu, KHV, CDX, or other sampled groups cannot be interpreted as definitive ancestry or ethnic identity. Fourth, centroid rankings depend on the number of principal components summarized, so interpretation emphasized patterns consistent across higher-dimensional summaries and the unpruned FST analysis. Fifth, reference-panel absence is conditional on the composition, genomic coverage, processing, and release of the HGDP–1000 Genomes dataset and cannot be interpreted as global novelty or population specificity. Sixth, the recurrent-variant screen is restricted by exome capture, the small cohort, genotype-quality thresholds, transcript choice, and the incompleteness and evolution of public clinical annotations; all prioritized variants therefore require independent technical validation and comparison with larger regional cohorts.

Future work should expand sampling across additional Southern Thai and neighboring Malaysian populations and should incorporate genome-wide reference panels representing Mainland Southeast Asian, Malay Peninsula, and Island Southeast Asian diversity. Integration with Thai-specific exome or genome resources, Pan-Asian SNP datasets, GenomeAsia, SG10K [41], and other Malay Peninsula reference panels would help refine the population placement of the Che He cohort beyond the currently available 1000 Genomes framework [3,4,8,19,40].

## 5. Conclusions

Whole-exome sequencing of 24 participants from Che He Subdistrict generated a technically robust exome dataset with high manufacturer-defined Agilent V8 target coverage and no evidence of close relatedness. All Che He samples projected within the East Asian space defined by 3380 high-quality unrelated HGDP–1000 Genomes references. Expanded-reference unpruned FST was lowest versus Cambodian, followed by Dai, KHV, CDX, and Lahu. These concordant findings provide a localized genomic reference while remaining descriptive of the available comparison panel rather than establishing ancestry or ethnicity. The reference-panel-absent analysis additionally provides a conservative, quality-controlled set of recurrent variants for future validation, but it should not be interpreted as evidence of private, globally novel, or pathogenic variation.

## Figures and Tables

**Table 1:** Study cohort, sequencing, preprocessing, and alignment summary.

Metric	Result
Participants included	24
Recorded sex	17 female (70.8%); 7 male (29.2%)
Age, mean ± SD	45.0 ± 12.1 years
Age, median (IQR)	47.0 years (34.25–49.25)
Age range	25–69 years
Raw reads per participant	107.7–127.7; mean 115.6 million
Raw sequence yield per participant	16.27–19.28; mean 17.46 Gb
Raw Q30 bases	92.14–93.66; mean 92.80%
GC content after filtering	47.04–49.53; mean 48.31%
Reads retained after fastp	106.6–126.2; mean 114.5 million
Bases retained after fastp	98.35–98.77; mean 98.57%
Post-filtering Q30 bases	92.60–94.05; mean 93.23%
Mapping rate to GRCh38	99.98–99.99; mean 99.99%
Properly paired reads	97.61–99.45; mean 98.73%
Picard duplicate rate	11.98–19.31; mean 15.42%
Mean depth across manufacturer-defined Agilent V8 targets	99.23–138.34; mean 117.07×

Note: Detailed alignment, duplicate, and insert-size metrics and summary manufacturer-target coverage metrics are provided in Supplementary Tables S2 and S3.

**Table 2:** Alignment and manufacturer-defined Agilent V8 target-coverage summary for 24 whole-exome samples.

Metric	Range	Mean
Final BAM reads per sample	65.1–89.1 million	76.9 million
Mapping rate	99.98–99.99%	99.99%
Properly paired reads	97.61–99.45%	98.73%
Picard duplicate rate	11.98–19.31%	15.42%
Mean insert size	240.0–264.7 bp	254.3 bp
Average base quality in BAM	35.6–37.3	36.6
Manufacturer-defined Agilent V8 target span	35,130,788 bp	—
Mean depth across manufacturer-defined Agilent V8 targets	99.23–138.34×	117.07×
SAMtools mismatch error rate	0.00212–0.00266	0.00239
Target bases covered at ≥10×	—	98.45%
Target bases covered at ≥20×	—	97.93%
Target bases covered at ≥30×	—	97.27%
Target bases covered at ≥50×	—	94.82%

**Table 3:** Variant discovery and PLINK quality-control summary.

Metric	Value
PASS cohort variants	421,801
PASS SNPs	360,916
PASS indels	60,885
Transition/transversion ratio	2.08
PLINK autosomal biallelic SNPs	351,623
Variants after WES-adjusted QC	226,348
LD-pruned SNPs	16,185
Samples retained	24/24
PASS variants within manufacturer-defined targets	69,512
Target-restricted PASS SNPs/indels	67,311/2201
Target-restricted autosomal biallelic SNPs before QC	65,764
Target-restricted autosomal biallelic SNPs after QC	63,632
Target-restricted samples retained	24/24
Post-QC target-SNP missingness	mean 0.0779%; median 0.0739%; range 0.0456–0.1666%

Note: PASS, variants that passed the applied variant filters; SNP, single-nucleotide polymorphism; QC, quality control; WES, whole-exome sequencing; LD, linkage disequilibrium.

**Table 4:** Internal cohort relatedness, missingness, heterozygosity, and PCA summary.

Metric	Result	Interpretation
Pairwise comparisons	276	All unique pairs among 24 participants
KING-robust kinship range	−0.1356 to 0.0002	No close relatives detected
Maximum kinship pair	C44_1E1-C01_1E1; 0.0002	Far below close-relative thresholds
Pruned SNP missingness per sample	1.12–1.80%	Low after LD pruning
Inbreeding coefficient F	−0.0163 to 0.1324	Exploratory heterozygosity metric
PC1 variance within cohort	5.29%	Internal PCA only; not ancestry inference
PC2 variance within cohort	5.24%	Internal PCA only; not ancestry inference

**Table 5:** Preliminary EthSEQ classification summary.

Measure	Result	Percentage/Range
Total participants	24	100%
INSIDE East Asian boundary	1	4.2%
CLOSEST to East Asian	23	95.8%
EAS relative contribution among CLOSEST samples	23 samples	68.94–83.84%
SAS relative contribution among CLOSEST samples	23 samples	16.16–31.06%

Note: EAS, East Asian superpopulation; SAS, South Asian superpopulation.

**Table 6:** Reference-panel PCA dataset and centroid-proximity summary.

Metric	Value
High-quality unrelated 1000 Genomes references	2510
High-quality unrelated HGDP references	870
Total reference individuals	3380
Che He individuals projected	24
Total projected individuals	3404
Exact allele-matched shared SNPs	297,205
SNPs after per-group missingness ≤ 5%	183,930
SNPs after reference-only MAF ≥ 1%	145,291
Reference-derived LD-pruned PCA SNPs	43,353
Post-filter mean missingness: 1KG/HGDP/Che He	0.0272%/0.1525%/0.1817%
Closest centroids over PC1–10	Cambodian, Lahu, KHV

Note: KHV, Kinh in Ho Chi Minh City, Vietnam; Cambodian and Lahu, HGDP population labels. Distances describe relative proximity within the available HGDP–1000 Genomes reference panel and do not constitute ancestry or ethnicity assignments.

**Table 7:** ADMIXTURE analysis summary for the merged Che He and 1000 Genomes dataset.

Metric	Value
Merged samples used for ADMIXTURE	2572
Che He samples	24
LD-pruned SNPs	43,328
K range evaluated	2–10
Cross-validation folds	10
Lowest CV-error model	K = 9
Minimum CV error	0.40039
CV error at K = 8/K = 9/K = 10	0.40045/0.40039/0.40044
Nearest 1KG population mean in K = 9 component space	KHV (EAS)

**Table 8:** Lowest ten unpruned pairwise Weir–Cockerham FST estimates between Che He and expanded HGDP–1000 Genomes reference populations, with exact Agilent V8 target-restricted sensitivity estimates.

Population	Primary FST (Approx. 95% Interval)	Primary Sites	Exact Agilent V8 FST
Cambodian	0.00256 (0.00157–0.00355)	179,678	0.00226
Dai	0.00892 (0.00754–0.01030)	179,574	0.00900
KHV	0.00956 (0.00892–0.01020)	180,089	0.00932
CDX	0.01149 (0.01081–0.01218)	179,883	0.01130
Lahu	0.01202 (0.01014–0.01391)	179,559	0.01166
Tujia	0.01259 (0.01138–0.01380)	179,598	0.01293
Miao	0.01382 (0.01247–0.01518)	179,619	0.01443
Naxi	0.01459 (0.01315–0.01603)	179,610	0.01477
Tu	0.01461 (0.01325–0.01597)	179,736	0.01410
Xibo	0.01509 (0.01360–0.01657)	179,757	0.01548

## Data Availability

Individual-level whole-exome sequencing reads and genotype data are not publicly available because they contain potentially identifiable human genomic information and public deposition was not covered by the original participant consent. Access to individual-level data may be considered upon reasonable request to the corresponding authors, subject to approval by the Human Research Ethics Committee, Faculty of Medicine, Prince of Songkla University, and applicable institutional data-governance requirements. The accompanying de-identified reproducibility package, containing aggregate results, analysis scripts, software and environment specifications, reference-source and filtering documentation, checksums, and selected aggregate results and supporting analysis files, is available from Zenodo: https://doi.org/10.5281/zenodo.22699278.

## Supplementary Materials

The following supporting information can be downloaded at: https://www.mdpi.com/article/10.3390/life16101651/s1, Supplementary Table S1: Raw-read, fastp, and post-trimming quality-control metrics for all 24 participants; Supplementary Table S2: Alignment, duplicate, insert-size, and general BAM metrics generated from SAMtools and GATK duplicate metrics; Supplementary Table S3: Summary of manufacturer-defined Agilent V8 target-coverage metrics generated using mosdepth; Supplementary Table S4: Variant-calling, hard-filtering, PLINK quality-control, and LD-pruning summary; Supplementary Table S5: Sample-level raw and pruned missingness, heterozygosity, and inbreeding estimates; Supplementary Table S6: Pairwise KING-robust kinship coefficients for all participant pairs; Supplementary Table S7: Internal cohort PCA coordinates; Supplementary Table S8: Summary of the expanded HGDP–1000 Genomes reference composition and cohort integration; Supplementary Table S9: Group-specific missingness before and after the ≤5% per-group variant filter; Supplementary Table S10: Summary of the reference-defined PCA projection of the HGDP–1000 Genomes reference and Che He samples; Supplementary Table S11: Summary of reference-population centroid proximity to the Che He centroid across PC1–4, PC1–10, and PC1–20; Supplementary Table S12: ADMIXTURE cross-validation errors for K = 2–10; Supplementary Table S13: ADMIXTURE K = 9 Che He individual ancestry-component proportions; Supplementary Table S14: ADMIXTURE K = 9 Che He component summary and nearest 1000 Genomes population means; Supplementary Table S15: Unpruned pairwise Weir–Cockerham FST estimates for Che He versus all 78 expanded HGDP–1000 Genomes reference populations, including exact Agilent V8 target-restricted sensitivity estimates and population ranks; Supplementary Table S16: Comparison of the manufacturer-defined Agilent V8 target intervals with the broader GENCODE v47 protein-coding exon plus 100-bp calling space; Supplementary Table S17: Summary of manufacturer-target-restricted variant counts, quality-control retention, and sample-level missingness; Supplementary Table S18: High-confidence recurrent variants observed in at least three Che He participants and absent from the expanded HGDP–1000 Genomes comparison panel after exact-source multiallelic reconciliation, with genotype-quality metrics and exploratory Ensembl VEP annotations; Supplementary Figure S1: Four-stage bioinformatics workflow for whole-exome sequencing analysis, from raw-read quality control through cohort variant calling and reference-panel population-genetic analyses. The broader GENCODE v47 protein-coding exon plus 100-bp intervals were used as standardized calling regions, while the exact manufacturer-defined Agilent V8 target intervals were used for coverage assessment and target-restricted sensitivity analysis; Supplementary Figure S2: Internal principal-component analysis of 24 Che He participants based on 16,185 LD-pruned autosomal SNPs. This analysis was used for within-cohort quality assessment and should not be interpreted as reference-based ancestry inference.
