# Supplementary material for: Genetic Ancestry and Affinity of a Local Population in Southern Thailand Using Whole-Exome Sequencing
Source: Life (Basel). 2026 Sep 30;16(10):1651. doi: 10.3390/life16101651 (PMC13705770; doi:10.3390/life16101651)
Supplement: Supplementary file 1 [file life-16-01651-s001.zip › Che_He_WES_05_Supplementary_Figures_S1-S2_2026-09-11.pdf]

Supplementary Figures

Genetic Ancestry and Affinity of a Local Population in Southern Thailand Using Whole-Exome Sequencing

Supplementary Figures S1–S2

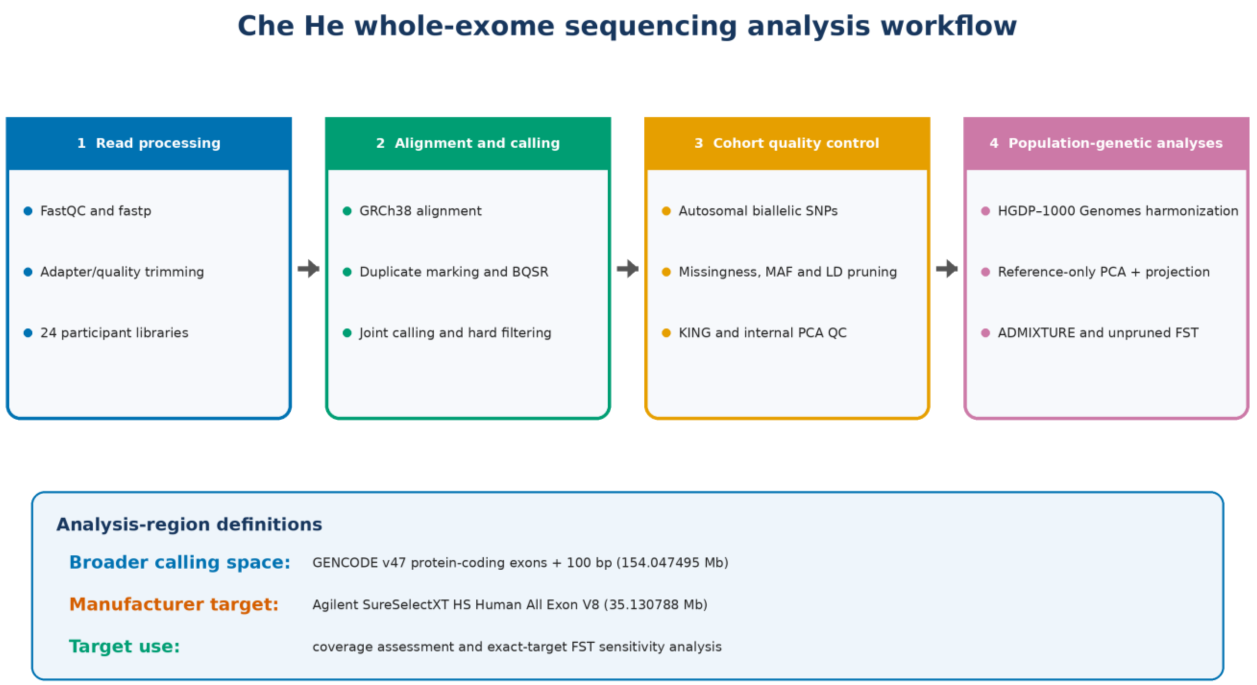

Supplementary Figure S1. Four-stage bioinformatics workflow for whole-exome sequencing analysis, from raw-read quality control through cohort variant calling and reference-panel population-genetic analyses. The broader GENCODE v47 protein-coding exon plus 100-bp intervals were used as standardized calling regions, while the exact manufacturer-defined Agilent V8 target intervals were used for coverage assessment and target-restricted sensitivity analysis.

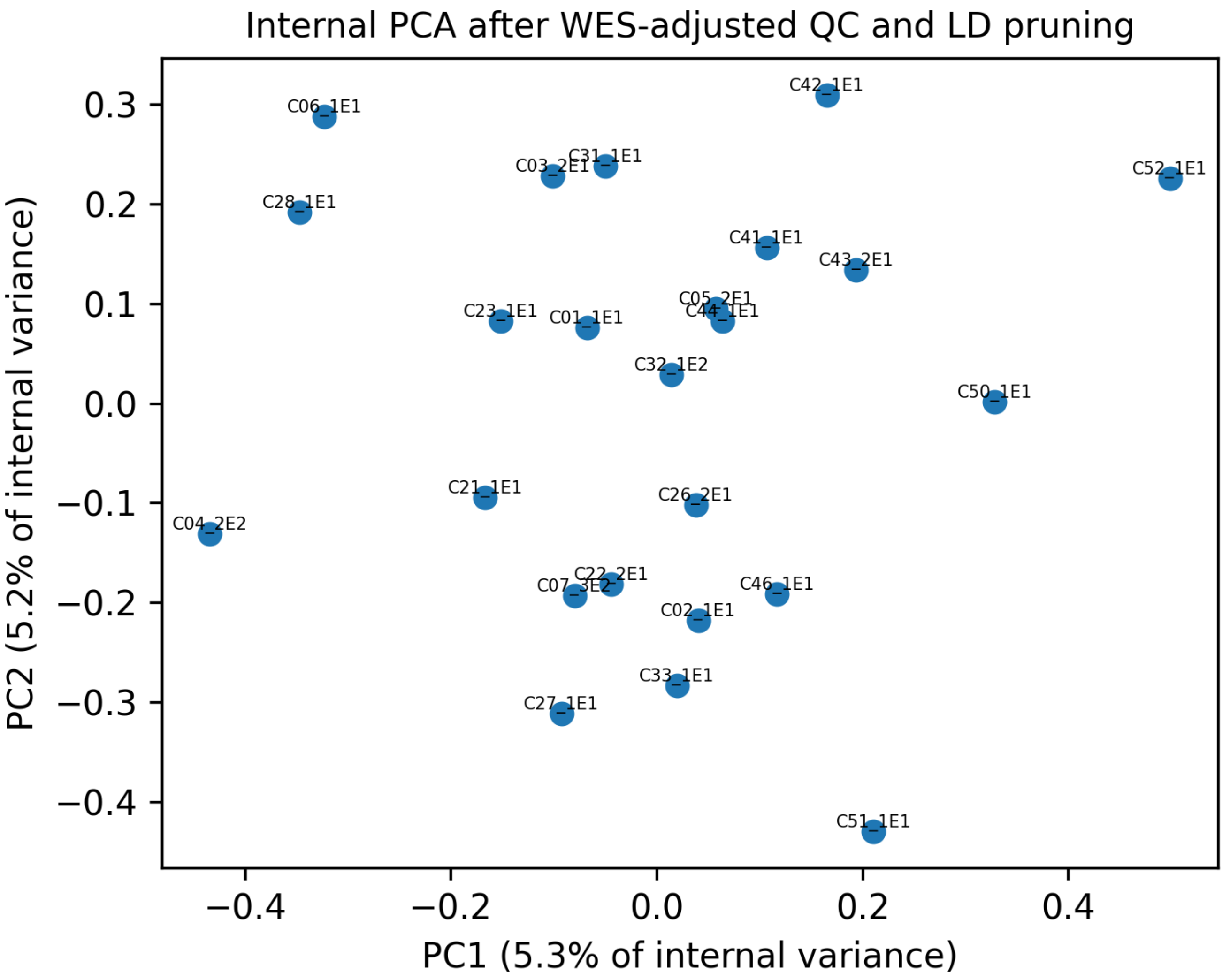

Supplementary Figure S2. Internal principal-component analysis of 24 Che He participants based on 16,185 LD-pruned autosomal SNPs. This analysis was used for within-cohort quality assessment and should not be interpreted as reference-based ancestry inference.
